# Spectroscopic investigation of defect-state emission in CdSe quantum dots

**DOI:** 10.3906/kim-2101-66

**Published:** 2021-06-30

**Authors:** Gülhan GÜLEROĞLU, Caner ÜNLÜ

**Affiliations:** 1 Department of Chemistry, Faculty of Arts and Science, İstanbul Technical University, İstanbul Turkey; 2 Department of Nanoscience and Nanoengineering, İstanbul Technical University, İstanbul Turkey; 3 İstanbul Technical University Nanotechnology Research and Application Center (ITUNano), İstanbul Technical University, İstanbul Turkey

**Keywords:** Quantum dots, surface defect emission, two-phase synthesis method

## Abstract

CdSe quantum dots are the most studied Cd-based quantum dots with their high quantum yield, high photostability, narrow emission band, and easy synthesis procedure. They are frequently used to develop light emitting diode (LED) due to their unique photophysical properties; however, their narrow emission band causes a challenge to design white LEDs because white light emission requires emission in multiple wavelengths with broad emission bands. Here in this study, we developed CdSe quantum dots with a narrow band-edge emission band and broad defect-state emission band through a modified two-phase synthesis method. Our results revealed that defect-state emission is directly linked to the surface of quantum dots and can be excited through exciting surfactant around the quantum dot. The effect of surfactant on emission properties of CdSe quantum dots diminished upon growing a shell around CdSe quantum dots; as a result, surface-dependent defect-state emission cannot be observed in gradient heterogeneous alloyed CdS_x_Se_1-x _quantum dots.

## 1. Introduction

Cadmium chalcogenide (Cd-chal) quantum dots (QDs) are the earliest quantum dots which have been synthesized and explored since the beginning of 1990s [1,2]. They are semiconductor materials with fluorescence properties and possess unique photophysical and structural characteristics such as high quantum yield, high photostability, single narrow emission band, wide absorption bands, high molar extinction coefficient, small size (2–10 nm), semiconductor nature, and modifiable surface [1–4]. With their unique properties, Cd-Chal QDs have been widely used in many different technologies such as solar cells, LEDs, biotechnology, military, and medicine [5–11]. Since they have excellent photophysical properties, they are frequently used in LED and solar cell applications [8,9] and even high-tech brands, such as Samsung, have adapted QDs into their monitor systems [12,13]. 

However, one of these excellent properties of QDs, i.e. having a narrow emission band, can be considered a disadvantage in white light emitting diode (wLED) design. In order to overcome this disadvantage, wLEDs with multiple quantum dot layers, which emit light in different colors, have been produced [14]. However, another unique feature of QDs that complicates design of multilayered QD films is their high molar extinction coefficient [1,2]. With high molar extinction coefficient, green light emissive QDs strongly absorb light in blue light spectrum [1,2] which are the two main colors in wLED design [14]. 

To prevent self-absorption of QDs in multilayered QD films, multicolor light emitting QDs with single composition have been proposed [3,15–21]. Two general ways to design multicolor light emitting QDs have been proposed: 1) doping binary or ternary QDs with an extra element [3,15–19], 2) creating surface defects [20,21]. Both of these methods cause formation of defect states and thus formation of defect-state emission in QDs’ emission spectrum. In a recent study, Ünlü et al. doped CdSe quantum dots with Te through a modified two-phase synthesis method and obtained controllable defect-state emission due to Te doping [3]. In a different study, Yuan et al. synthesized nitrogen-doped carbon quantum dots to achieve white light emission through a single-component system [15]. Yang et al. synthesized dual emissive Ag:InP/ZnS quantum dots through a hot injection method and achieved high quantum yield around 75% [16]. Zhang et al. developed Mn-doped InP/ZnS quantum dots via a growth-doping method for wLED design [17]. Budak et al. synthesized boron- and nitrogen-doped graphene QDs through a hydrothermal synthesis method and observed a controllable dual emission [18]. Again, Zhang et al. synthesized Cu:InP/ZnS through a growth-doping method and achieved dual emissive QDs with quantum yield around 75% [19]. Samuel et al. achieved to create surface-dependent emission in CdSe quantum dots through a surface reconstruction method and as a result increased the quantum yield of QDs by 7% [20]. Moreover, Samuel et al. explored the potential of CdSe QDs with surface defect emission for wLED design [21]. However, so far, defect states on CdSe QDs have never been created through two-phase synthesis method, the method which takes place at ambient conditions and results in high quantum yield [3,4,22–24]. 

In this study, we synthesized CdSe quantum dots with high defect-state emission through a modified two-phase synthesis method. The synthesis was carried under ambient pressure with an inert gas atmosphere at mild temperature. The CdSe QDs synthesized in this work possessed 2 emission bands; one emission band belonged to band-edge emission and the other belonged to defect-state emission. The defect-state emission disappeared as the CdSe QDs were covered with CdS layer. The defect-state emission could be triggered through excitation of surfactant of CdSe quantum dot. This study is the first to examine defect-state emission in CdSe QDs which are synthesized through a two-phase synthesis method and correlates the excitation of defect-state emission directly to the surfactant, tri octylphosphine oxide. 

## 2. Materials and methods

All chemicals that were used in this work were of the highest purity and were purchased from the Sigma-Aldrich Co. They were used without further purification.

### 2.1. Synthesis of cadmium myristate

Cadmium myristate was synthesized through a general procedure reported previously in the literature [3,4]. Firstly, 1.28 g (10 mmoles) of cadmium oxide (CdO) was mixed with 4.56 g (20 mmoles) of myristic acid and then the mixture was heated at 210 °C for 15 min under inert gas (nitrogen) atmosphere. Formation of cadmium myristate (CdMA) was identified as brownish CdO–myristic acid mixture became a colorless liquid and bubbling out of O_2_(g), which is the by-product, stopped. CdMA was purified through recrystallization in toluene, and obtained CdMA solid was dried and stored at 4 °C for further experiments.

### 2.2.Synthesis of sodium hydrogen selenide

Sodium hydrogen selenide (NaHSe) was synthesized through a general procedure reported previously in the literature [3,4]. Firstly, 10 mg of Se was put in a 100-mL reaction flask, sealed with a plastic plug and nitrogen gas was purged into a sealed reaction flask to remove oxygen completely. Then, 15 mg of sodium borohydride (NaBH_4_) was dissolved in 10 mL of ultrapure water and bubbled with nitrogen gas. NaBH_4_ solution was then mixed with Se under an inert gas atmosphere. The mixture was heated to 60 °C to speed up the reaction. The reaction was stopped as Se solid completely disappeared in solution and bubbling out of H_2_(g), which is the by-product, finished. NaHSe was prepared freshly for each experiment.

### 2.3. Synthesis of CdSe quantum dots with high amount of defect states

The CdSe quantum dots were obtained through a modified two-phase synthesis method [3,4]. Firstly, 0.1 g of CdMA and 1.5 g of trioctyl phosphineoxide (TOPO) were mixed in 45 mL of toluene at 80 °C until they were completely dissolved under an inert gas atmosphere. After CdMA and TOPO were dissolved, reaction temperature was set to 100 °C and 42 mL of ultrapure water was added to the system and the two-phase reaction mixture was kept stirring for 5 min under an inert gas atmosphere to stabilize the temperature of the reaction system. Then, 3 mL of NaHSe solution (3 mg NaHSe in total) was added to the reaction system and CdSe quantum dot formation began. Aliquots were taken from toluene to monitor the emission properties of the quantum dots by using fluorescence spectrophotometer. The reaction was stopped by cooling the solution to the room temperature at the 3rd h after NaHSe injection. The quantum dots were purified through precipitation by addition of excess methanol to toluene. This synthesis procedure was repeated to check repeatability.

### 2.4. Synthesis of CdSxSe1-x quantum dots

CdS_x_Se_1-x_ quantum dots were synthesized through a general procedure proposed in the literature before. In short, the same procedure used in the synthesis of CdSe quantum dots was performed with a slight modification; 60 mg of thiourea dissolved in 42 mL of ultrapure water was added together with 3 mL of NaHSe to reaction system in order to start growth of CdS_x_Se_1-x_.

### 2.5. Optical characterization of CdSe and CdSxSe1-x quantum dots

Optical properties of CdSe and CdS_x_Se_1-x_ quantum dots were checked using an ultraviolet–visible (UV–Vis) spectrophotometer and a fluorescence spectrophotometer. Absorption spectra of quantum dots were collected using a Scinco Neosys-2000 double-beam UV–Vis spectrophotometer. Emission and excitation spectra of nanocrystals were collected using a Varian Cary Eclipse fluorescence spectrofluorometer. Emission spectra of CdSe and CdS_x_Se_1-x_ quantum dots were recorded by using excitation wavelength (λ_exc_) 350 nm. Each sample was dissolved in 10 mL of toluene after purification and diluted by 10 factor before characterization in order to prevent errors resulting from self-absorption. The same samples were used to collect the excitation spectrum. Excitation spectra of CdSe quantum dots were collected at two wavelengths; 450 nm and 580 nm. Excitation spectra of CdS_x_Se_1-x_ quantum dots were collected at 525 nm.

## 3. Results and discussion

### 3.1. Synthesis of CdSe and CdS_x_Se_1-x_ quantum dots

**Figure 1:**
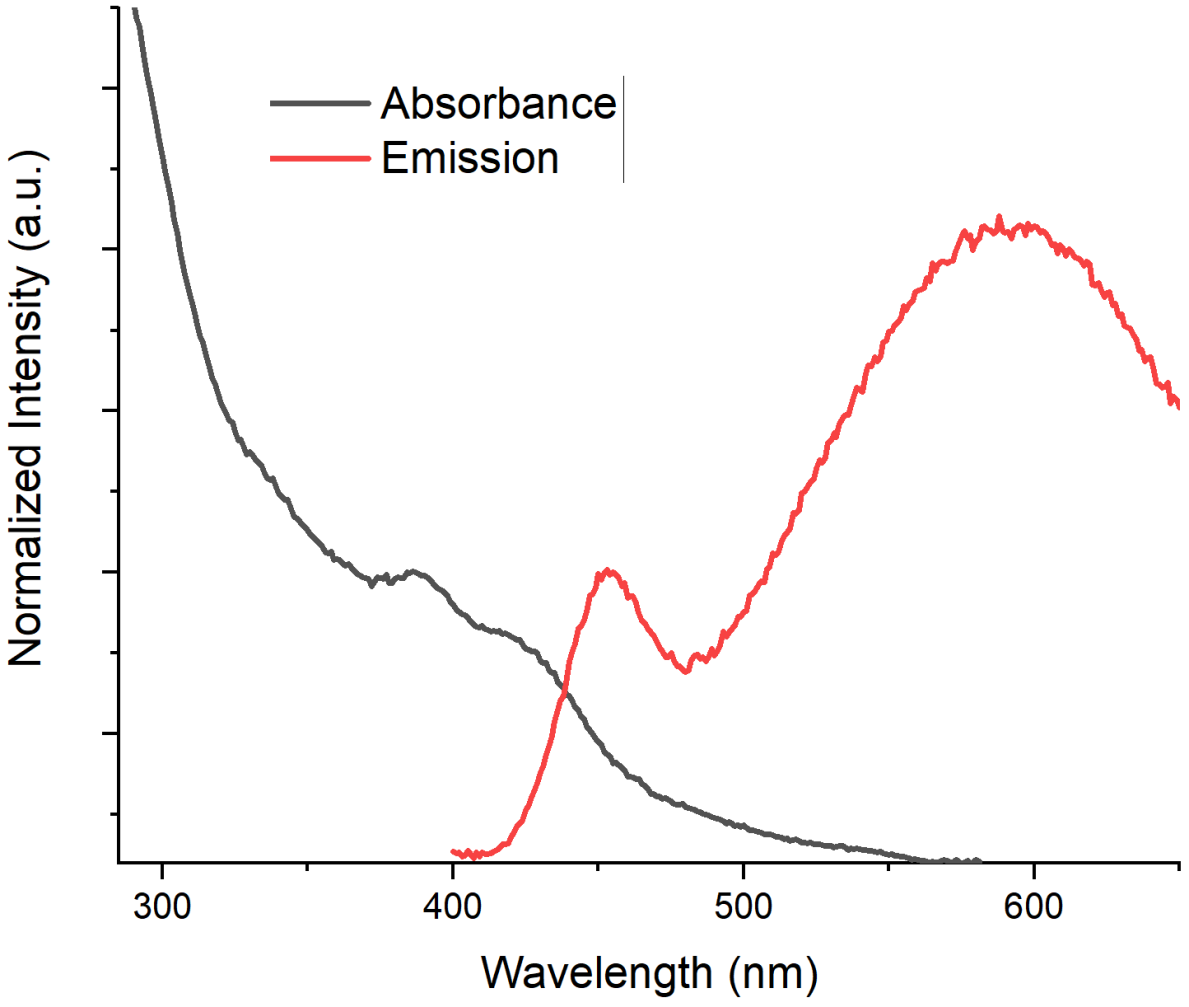
Absorption (Black) and emission spectra (Red, λexc= 350 nm) of CdSe QDs. The intensity of absorption spectrum is normalized to 1 with respect to the intensity at 380 nm. The intensity of emission spectrum is normalized to 1 with respect to the intensity at 460 nm.

**Figure 2:**
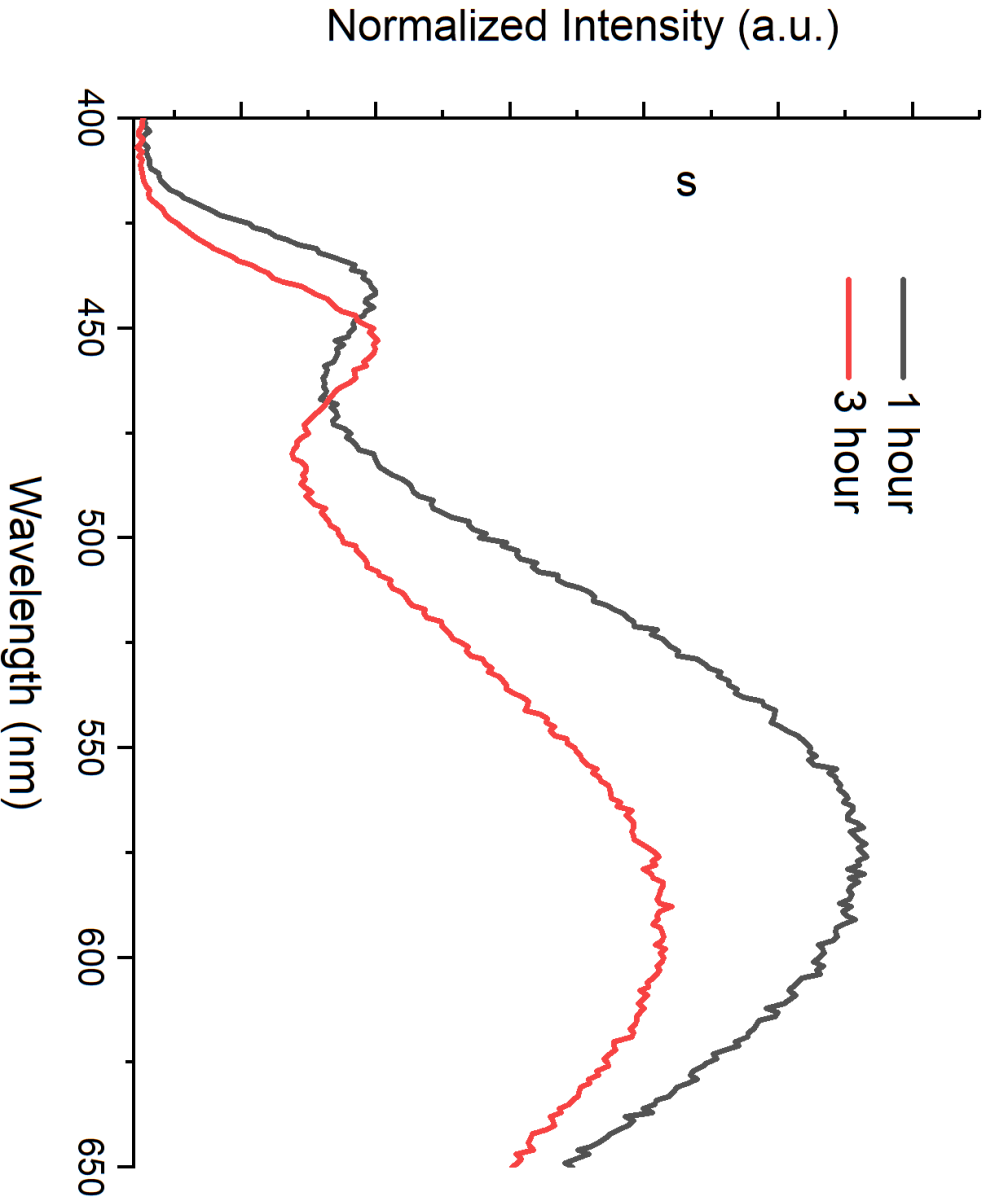
Emission spectrum of CdSe QDs with defect states at 1 h (black) and at 3 h (red). The intensity of each spectrum was normalized to 1 with respect to the intensity of the first peak.

**Figure 3:**
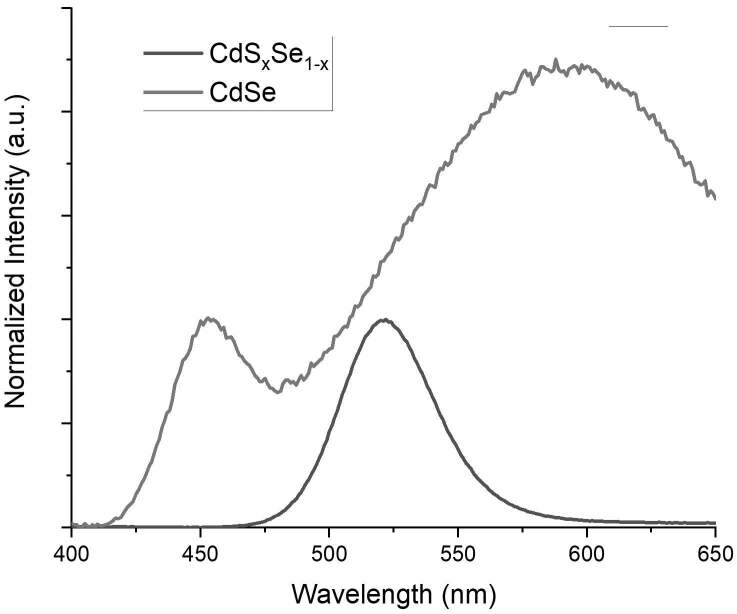
Emission spectrum of CdSe (Red) and CdSxSe1-x (Black) QDs with λexc=350 nm. The intensity of each spectrum was normalized to 1 with respect to the intensity of the first peak.

Both CdSe and CdS_x_Se_1-x_ quantum dots were synthesized through a modified two-phase approach [3,4,25]. In traditional two-phase synthesis approach, Cd precursor is dissolved in the nonpolar phase (toluene) together with surfactant where chalcogen precursors are dissolved in the polar (aqueous) phase. The quantum dots form at the interface of the two phases and dissolve in the nonpolar phase. Compared to traditional solvothermal synthesis methods, the two-phase synthesis method offers more control on size of the quantum dots since the reaction time is lengthened out considerably. Generally, in the two-phase synthesis method, synthesis of CdSe quantum dots takes place in a sealed hydrothermal reaction vessel in order to minimize formation of defect states [22,23,26]. In 2013, Ünlü et al. proposed a modified two-phase approach to synthesize CdS_x_Se_1-x_ quantum dots with heterogeneous–gradient structure in a 3-necked reaction vessel under nitrogen atmosphere without sealing and creating pressurized synthesis conditions [4]. By creating a heterogeneous–gradient structure, Ünlü et al. managed to synthesize core-shell like alloyed CdS_x_Se_1-x_ quantum dots with very high quantum yield (up to 90%) by using oleic acid or TOPO as surfactant [4]. As a consequence, CdS_x_Se_1-x_ quantum dots had a single very narrow emission band with almost no defect-state emission [4]. However, in Ünlü et al.’s study, it should be noted that using oleic acid instead of TOPO as surfactant brought out two important consequences: 1) As oleic acid was used as surfactant, the reaction slowed down dramatically, 2) The quantum yield of the CdS_x_Se_1-x_ quantum dots dropped to 50% as TOPO was used as a surfactant [4]. Both of these outcomes showed that using TOPO as a surfactant yields an increase in formation of defect states [4]. In the current study, CdSe quantum dots were synthesized in a 3-necked reaction vessel without sealing and creating pressurized synthesis conditions by using TOPO as the surfactant. By doing so, CdSe quantum dots were formed with a high amount of defect state, which could be observed in the emission spectrum of CdSe quantum dots (Figures 1 and 2). The defect-state emission disappeared in the emission spectrum of CdS_x_Se_1-x_ quantum dots (Figure 3) as a result of core-shell like gradient alloyed structure.

### 3.2. Absorption and emission properties of CdSe quantum dots

Absorption spectrum of CdSe quantum dots displayed typical features of CdSe quantum dots (Figure 1) [3,4,25]. CdSe absorption spectrum had 2 shoulders at 390 nm and 420 nm (Figure 1) due to formation of CdSe quantum dots, a typical feature of the absorbance spectrum of CdSe quantum dots [3,4,25]. As another typical feature of quantum dot absorption, CdSe absorption spectrum possessed an intensive band below 300 nm (Figure 1) [3,4,25]. Emission spectrum of CdSe quantum dots had two intense bands; one narrow band with a peak at 450 nm (band-edge emission) and one broad band with a peak at 580 nm (defect-state emission) (Figure 1). 

The peak position of the band-edge emission band shifted towards the low energy region with increase in time, which is due to increase in size of quantum dots (Figure 2). The peak point of defect-state emission shifted towards the low energy region, too. However, the relative intensity of defect-state emission significantly decreased with increase in reaction time (Figure 2). As these results were compared with results of a previous work published by Ünlü et al., which was about controlling defect-state emission in Te-doped CdSe, the outcomes were completely different [3]. In the aforementioned work, CdSe quantum dots were doped with Te atoms in the presence of oleic acid as surfactant and defect-state emission was observed as a result [3]. The peak point of defect-state emission in Te-doped CdSe quantum dots was steady, did not shift by reaction time, and became more intense with increase in reaction time and with increase of Te ratio in synthesis medium [3], which showed that Te doping is the main reason behind formation of defect states in Te-doped CdSe quantum dots. As the results of current study were compared with those of Ünlü et al.’s work [3], it was observed that the origin of defect-state emission in TOPO-capped CdSe quantum dots was completely different from that of oleic-acid-capped Te-doped CdSe quantum dots. 

In order to control the synthesis conditions of CdSe quantum dots, a parallel synthesis was performed with the same conditions to synthesize CdS_x_Se_1-x_ quantum dots. The emission spectrum of CdS_x_Se_1-x_ quantum dots had a single, narrow emission band with a peak point at 525 nm (Figure 3). The complete disappearance of defect-state emission was a result of core-shell like structure of CdS_x_Se_1-x_ quantum dots [4]. As the rapidly formed CdSe-rich inner structure was covered with a slow growing CdS outer shell, the defect-state emission disappeared (Figure 3.). This result indicated that as the defected core is surrounded by an extra inorganic slow-growing shell, the amount of surface-based defects decreased; as a result, defect-state emission could no longer be observed (Figure 3).

### 3.3. Origin of defect-state emission in CdSe quantum dots

In order to understand the difference between characteristics of defect-state emission and band-edge emission, photoluminescence excitation (PLE) spectra of CdSe quantum dots at 455 nm and 580 nm were collected (Figure 4). PLE spectra of CdSe quantum dots at 455 nm and 580 nm had both similarities and differences. Both spectra had 3 peaks at 420 nm, 390 nm, and 330 nm. The peaks at 390 nm and 420 nm were also observed in the absorbance spectrum of CdSe quantum dots, which shows that both band-edge emission and defect-state emission can be excited at these wavelengths. The peak at 330 nm in PLE spectra of CdSe quantum dots at 455 nm and 580 nm revealed that both band-edge and defect-state emission could be excited with similar wavelengths, because all of these excitation bands belonged to the same structure, the CdSe quantum dots. The peak at 290 nm in PLE spectrum of CdSe quantum dots at 580 nm is due to harmonic wave of 580 nm emission and did not occur due to emission properties of CdSe quantum dots, but occurred due to emission wavelength which was chosen by user [3,4,25]. However, the peak at 260 nm in the PLE spectrum of CdSe quantum dots at 580 nm completely disappeared in the PLE spectrum of CdSe quantum dots at 455 nm (Figure 4). In the absorbance spectrum of CdSe quantum dots, the peak at 260 nm was very intense, as was observed in different types of quantum dots in the literature as a common feature [3,4,25]. However, this peak does not contribute to fluorescence of quantum dots and is attributed to nonradiative electron transitions and background materials such as surfactants [3,4,25]. According to the results shown in Figure 4, the band-edge emission could not be excited by 260 nm; however, the defect-state emission could be excited. This result indicated that the defect-state emission in CdSe quantum dots was derived from the nanocrystal structure, but defect-state emission could also be obtained through excitation of surfactant which covered the inorganic CdSe core. The defect states on the surface of CdSe quantum dot are the main factor that clarifies the defect-state emission of the CdSe quantum dots.

To understand the effect of surface defects on defect-state emission, CdS_x_Se_1-x_ quantum dots were synthesized. As was discussed before, alloyed CdS_x_Se_1-x_ quantum dots have heterogeneous–gradient structure with CdSe rich inner core covered with CdS rich outer shell [4]. The slow growth rate of CdS-rich outer shell minimizes the amount of surface defects; as a result, quantum dots with higher quantum yield and narrower band edge emission were obtained [4]. As the PLE spectra of CdS_x_Se_1-x_ quantum dots and CdSe quantum dots were compared, it was observed that there was no excitation peak in PLE spectrum of CdS_x_Se_1-x_ quantum dots at emission peak (525 nm) which was located in between band-edge emission (455 nm) and defect-state emission (580 nm) peaks of CdSe quantum dots (Figure 5). This result revealed that as the amount of defects on the surface of CdSe quantum dots were minimized, the defect-state emission disappeared.

## 4. Conclusion

CdSe quantum dots are the most studied quantum dots with their unique optical properties. However, most of the research on CdSe quantum dots focuses on band-edge emission properties of CdSe quantum dots. Here in this paper, we managed to synthesize CdSe quantum dots with two intense emission bands, band-edge emission and defect-state emission, through two-phase synthesis methods. Due to the relatively slow growth rate in the two-phase synthesis method, we were able to control the relative intensity of defect-state emission in CdSe quantum dots. Emission and PLE spectra of CdSe quantum dots revealed that the defect-state emission of CdSe quantum dots can be excited separately from band-edge emission and the origin of defect-state emission in TOPO-capped CdSe quantum dots solely depends on the defects on the surface of CdSe quantum dots.
